# Forcible Feeding and the Penal Reform League

**Published:** 1913-11-08

**Authors:** Arthur St. John

**Affiliations:** Hon. Sec. Penal Reform League. 68A Park Hill Road, N.W.


					forcible Feeding and the Penal Reform League.
To the Editor of The Hospital.
Sir,?With reference to reports which have appeared
the press announcing the resumption of forcible feed-
in prison, the committee of the Penal Reform League
desire me to draw your attention to a resolution passed at
their meeting in July last to the following effect:?" The
Executive Committee of the Penal Reform League, having
111 view the discredit brought on law by what is known
Popularly as the ' Cat and Mouse Act/ and the injury
lr>flicted thereby on the cause of penal reform, hereby
exPresses its conviction that when a prisoner who is in
Prison for conscience' sake brings himself by hunger-
'^riking or similar self-discipline to a state of health
^'hich, in the opinion of the medical officer of the prison
ln which he is confined, endangers his life, then justice
humanity demand that he be released unconditionally."
* V committee wish me to add that, in their opinion, even
s? grave a misfortune as the death of one or two prisoners
^v?uld be a less serious evil than the deterioration of the
floral sense of the community effected by acquiescence in
Su?h an abominable outrage as is involved in forcible
^e&ding.?I have the honour to be, Sir, your obedient
Servant, Arthur St. .Tonv.
Arthur bx. John,
Hon. Sec. Penal Reform League.
Park Hill Road, N.W.

				

